# Validation of a Measurement Scale on Technostress for University Students in Chile

**DOI:** 10.3390/ijerph192114493

**Published:** 2022-11-04

**Authors:** Alejandro Vega-Muñoz, Carla Estrada-Muñoz, Paola Andreucci-Annunziata, Nicolas Contreras-Barraza, Heidi Bilbao-Cotal

**Affiliations:** 1 Instituto de Investigación y Postgrado, Facultad de Ciencias de la Salud, Universidad Central de Chile, Santiago 8330507, Chile; 2 Public Policy Observatory, Universidad Autónoma de Chile, Santiago 7500912, Chile; 3 Departamento de Ergonomía, Facultad de Ciencias Biológicas, Universidad de Concepción, Concepción 4070386, Chile; 4 Facultad de Economía y Negocios, Universidad Andres Bello, Viña Del Mar 2531015, Chile; 5 Independent Researcher, Santiago 7500885, Chile

**Keywords:** technostress, information technology, behavior studies, overwork, mental health, higher education, students

## Abstract

The main aim in this research was to validate a scale for measuring technostress in Chilean university students under the context of hybrid education. There were 212 university students as participants from the central-south zone of Chile. For measuring technostress manifestations, a technostress questionnaire for Chinese university professors and its adaptation for Spanish university students was used as a base instrument to adapt the scale. The exploratory and confirmatory factor analysis generated an adequacy of the psychometric scale by eliminating three items from the original scales but generated important changes by reordering the other 19 items into only three factors, establishing an important local difference with previous versions that contemplated five factors, but retaining as a central axis the stress produced by a misfit between the person and his or her environment. The resulting scale was based on factors such as Abilities-Demands Techno-Educational, Needs-Supplies Resources, and Person-People Factor. It also has a good internal consistency with a scale that allows for the continuation of technostress measurements in the local context; adding to studies on this topic which have already been carried out on diverse actors of the Chilean educational system; proposing a reliable and valid psychometric scale of technostress in Chilean university students; and giving researchers and academic managers the ability to know the adverse effects of the use of technologies and propose mitigation actions.

## 1. Introduction

The incorporation of information and communication technologies (ICT) in education has changed the nature, methods, and processes of learning [1,2]. Nowadays, education has been restructured due to the increasing ICT usage rate by school and university students [3]. According to Talebian et al. [4], ICT enriches existing educational models and provides new technology-based training and learning schemes. At the higher educational level, technological development has facilitated the student exchange between universities and cooperation instances between international students [3]. Likewise, ICTs have challenged and accelerated technological skills development, stimulating “learning by doing” and contributing to sustainable development through the fourth sustainable development goal on quality education [5,6,7].

ICT use, however, can cause stress, which corresponds to a physical and emotional response to distress caused by an individual’s imbalance between perceived demands and perceived resources, and their abilities to cope with those demands [8]. When stress is associated with ICT use, it is called technostress, a concept first coined by the American psychiatrist Craig Brod [9], who defined it as a “modern adaptative illness caused by the inability to cope with new computer technologies in a healthy manner” [10] (p. 242). In the last two decades, it has become a topic of growing interest for studies [11,12].

Salanova defines technostress as “a negative psychological state related to the ICT use or threat of its use in the future, conditioned by the mismatch perception between the demands and resources related to the ICT use that leads to a high unpleasant psychophysiological activation level and to the negative attitude development towards ICT” [13] (p. 423). For its part, according to Tarafdar et al. [14], technostress is a product of the inability to adapt or cope with new technologies, and constitutes a process characterized by the presence of technological environmental conditions that are evaluated as demands or techno-stressors by the individual, which set in motion coping responses leading to psychological, physical, and behavioral manifestations.

Regarding technostress study fields, at the educational level, research predominates, above all, in primary and secondary education teachers [15,16,17,18,19], in university teachers [20,21,22,23], administrative workers [24,25], and librarians [26]. Even though they are scarce, studies on university students are also mentioned, which highlight that technostress significantly predicts burnout and negatively impacts academic productivity [27,28,29,30].

The new challenges resulting from the COVID-19 pandemic for higher education through ICT [31,32,33,34,35] and given the eruption of hybrid higher education [36,37], an interest in learning about its psychological and mental health effects on students is evident. Given this scarcity of studies on technostress in university students, it is necessary to have psychometric measurement scales adapted and validated to various local contexts to facilitate further study. In this regard, this article aimed to adapt and validate measurement of technostress scale for university students (TS4US) based on previous work by Wang et al. [21] in China, and Penado-Abilleira et al. [38] in Spain. In theoretical terms, this local validation contributes to strengthen the global empirical case set that allows to specify the theoretical constructs incorporated in a psychometric instrument capable of measuring technostress in university students.

In the Chilean case, technostress studies in education have been published in mainstream journals focusing on other members of educational communities, such as teachers [18,19] and managers [25]. In practical terms, this work will allow access to an instrument with structural validity to advance the study in university students.

## 2. Materials and Methods

The adaptation for Chilean students of the Wang and Li [21] and Penado-Abilleira et al. [23] technostress questionnaires began with a re-translation of the items that compose the original Wang and Li [21] scale to the Spanish of current use in Chile by a specialized linguist who was part of the research team, and adapting the items for a university student population as well as establishing local semantic differences in comparison with Penado-Abilleira et al. [23]. The result was pre-tested with a group of 20 students to evaluate their comprehension. The resulting scale is presented in Appendix A (in Chilean Spanish), keeping a 5-point Likert scale: Strongly Disagree = 1, Disagree = 2, Neither Disagree nor Agree = 3, Agree = 4, and Strongly Agree = 5. The questionnaire has been self-administered online, after giving informed consent response, collecting the survey without respondent identification, and only presenting non-potentially identifiable human data.

Then, SPSS 23 software was used (IBM, New York, NY, USA) to analyze the 22-item TS questionnaire [21,38], for establishing psychometric validity by means of structural evidence [39]. As a first step, a univariate descriptive statistical analysis was applied, with emphasis on variance (>0), skewness and kurtosis (|≤1|, both). To measure confidence levels, the authors applied the Kaiser-Meyer-Olkin measure of sampling adequacy (KMO). Moreover, the authors used Bartlett’s test of sphericity to identify items belonging to the factors within the scale as a form of exploratory factor analysis (EFA) with the extraction method, unweighted least squares (ULS), rotation method, and Oblimin with Kaiser normalization [40]. Then the authors analyzed the exploratory factors by using a confirmatory factor analysis (CFA) with FACTOR software [41]. In addition, they used the Hull method to select the number of factors according to the EFA results, including high communalities, high factor loadings to support sample size, and minimum items per factor (MIF) [42,43,44], considering the comparison possibilities with Wang et al. [21] and Penado-Abilleira et al. [38]. It was necessary to obtain a report of the indicators: Chi-square/degree freedom ratio (χ2/df), root mean square error of approximation (RMSEA), adjusted goodness-of-fit index (AGFI), goodness-of-fit index GFI, comparative fit index (CFI), (RFI), normed fit index (NFI), non-normed fit index (NNFI), and Root Mean Square of Residuals (RMSR) [45]. See Table 1.

Finally, the internal reliability of the resulting instrument will be validated by calculating Cronbach’s Alpha by SPSS 23 software [48,49].

### Sample Characterization

The Technostress Scale for University Students (TS4US) was applied in the first academic semester 2021 to a set of 212 participants (≥ 200, overcoming small sample sizes for factorial analysis) [43], university students from the Chile Central-South Zone, which concentrates more than 70 percent of the national and university population [50,51], as shown in Table 2 (Available data in Table S1: TS4US_data_22_var, Supplementary Materials).

## 3. Results

### 3.1. Exploratory Factor Analysis

**Figure 1:**
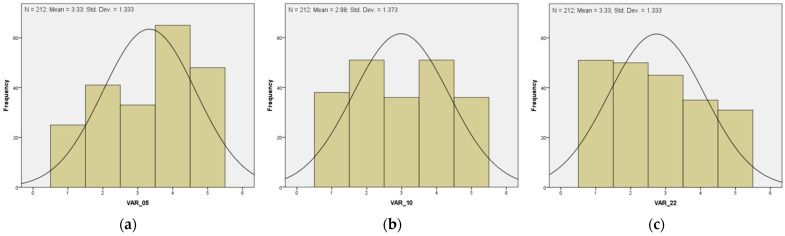
Excluded variable histograms: (**a**) VAR_05; (**b**) VAR_10; and (**c**) VAR_22.

Firstly, we analyzed the possible prevalence of the factors identified by Wang and Li [21] for Chinese university teachers and Penado-Abilleira et al. [38] for Spanish university students. These five original factors establish the stresses for technology use between personal capabilities and organizational demands (abilities-demands organization, ADO), personal capabilities and technology demands (abilities-demands technology, ADT), personal needs and organizational resources to perform their tasks (needs-supplies organization, NSO), personal needs and their own available technology resources (needs-supplies technology, NST), and interpersonal relationships between students (person-people factor, PPF). Univariate descriptive statistical analysis was applied, and no ordinal variable presented variance equal to zero, so all of them contributed to the common variance. But in terms of skewness and kurtosis, 3 variables present kurtosis (kurt) problems: VAR_05 (kurt = −1.109), VAR_10 (kurt = −1.281), and VAR 22 (kurt = −1.160), see Figure 1.

Table 3 and Table 4 show the unrestricted results of the exploratory factor analysis preserving the 18 variables and determining with SPSS 23 a KMO of 0.897 and Bartlett’s test with a Chi-square of 2432.170 with 171 degrees of freedom and a significance level of 0.000 for the four factors TS4US instrument. The authors achieved a 59.476% explained variance proportion. Although these results were individually valued as positive, it was also observed that factor 4 did not comply with the minimum variables recommended per factor (>3) [42,43,44], which led us to test another variable reduction alternative.

Table 5 and Table 6 show the result of the unrestricted exploratory factor analysis preserving 19 variables and determining identical results for KMO and Bartlett’s Test with the use of SPSS 23 for the three factors TS4US instrument and achieving a 54.264% explained variance proportion.

### 3.2. Confirmatory Factor Analysis

Additionally, the authors successfully adapted the 19 variables analyzed dataset for 19 variables to confirmatory factor analysis (CFA) with the use of the FACTOR software. The CFA obtained a KMO-Kaiser-Meyer-Olkin-equal to 0.89743 (>0.8) and Bartlett’s test of sphericity with a Chi-Square 9668.9 with 171 degrees of freedom and a significance level of 0.000010. Those results were significant and good enough to present the adequacy of the Pearson correlation matrix.

The Hull method for selecting the number of three factors was implemented with an adequacy of the Pearson correlation matrix. Then the authors reduced the TS4US questionnaire according to its latent variables in three factors (see Table 7).

Table 8 sets out the proposed model results in comparison with the resulting validity and reliability values in Wang et al. [21] and Penado-Abilleira et al. [38], for the RMSEA, AGFI, GFI, CFI and RMSR indicators by FACTOR software. In comparative terms, the proposed model reports an RMSEA with an acceptable fit equal to Wang et al. [21], AGFI and GFI with a good fit equal to Penado-Abilleira et al. [38], CFI with a good fit in contrast to the acceptable fit reported by Wang et al. [21], and RMSR with a good fit in contrast to the acceptable fit reported by Penado-Abilleira et al. [38].

Finally, Table 9 shows the instrument’s internal reliability by SPSS 23 software, with a total Cronbach’s Alpha of 92.5% for the set of 19 items, whose definitive scale is presented in Chilean Spanish in Appendix B.

## 4. Discussion

In this research, through an exploratory and confirmatory factor analysis, the factors identified by Wang and Li [21] for Chinese university teachers and Penado-Abilleira et al. [38] for Spanish university students were analyzed in the context of Chilean university students from public and private institutions. As a result of these analyses, within the Chilean university students participating in this study, the model explaining technostress was reduced from five to three factors, with the loss of three variables 5 (ADO05), 10 (ADT01), and 22 (PPF04), a product of the instrument’s adjustment to the specific sample composition; maintaining factors (theoretical constructs) that made it possible to measure the phenomenon of technostress. It was observed that the variables that make up the measurement scale tend to be grouped into the main factors that are part of various theories that explain stress, as detailed below.

The variables corresponding to the tensions between personal needs and organizational resources (NSO) and personal needs and technological resources (NST) are grouped in the first factor; it is known that the availability and usability of resources is a moderating factor on stress [52]. It should be noted that, in this factor, the variables that make up the personal needs or demands and technological resources (NST) that are associated with the self-perception of usefulness of available ICTs are grouped together, resulting in a factor on personal needs and available resources (needs-supplies resources, NSR). In this regard, the perceived usefulness of ICT use has been defined as a factor which inhibits technostress [53,54]. 

Then, there is a second factor, which groups those variables associated with interpersonal relationships (PPF) among students, maintaining this factor according to Wang and Li [21] and Penado-Abilleira et al. [38], which is associated with the social support given by peers and peer learning in the face of novel and potentially distressing processes [2,12], which constitutes a protective factor mitigating technostress [55]. 

Also, the third factor includes those variables associated with the relationship between personal capabilities and organizational demands (ADO), and personal capabilities and technological demands (ADT). If work demand is not in line with capabilities, this can lead to stress manifestations [56,57,58,59]. On the other hand, it includes variables on personal needs and technological resources (NST), but those associated with technological overload, which might be a cause for technostress rather than from the organizational type [9,60,61]. Because of high demand and lack of resources for working, ICT is associated with increased technostress [13,52]. In sum, the third factor relates personal capabilities and techno-organizational demands, specifically techno-educational demands (abilities-demands techno-educational, ADTE).

Finally, in the absence of a meta-analysis to ensure a broad application of a valid scale [62,63], the local validation is a contribution to the global empirical case set to clarify the theoretical constructs incorporated in a psychometric instrument with the ability to measure technostress in university students in different countries and cultures, as well as its practical implications in local terms to expand the technostress studies in university students.

## 5. Conclusions

This article validated a scale to measure technostress in Chilean university students in the context of hybrid education, based on activities combined and carried out in different environments (physical or virtual) and times (synchronous or asynchronous) [64,65], using as a basis a technostress questionnaire for Chinese university teachers and its adaptation for Spanish university students. The exploratory and confirmatory factor analyses eliminated three items from the original scales but generated important changes by reordering the other 19 items in only three factors, establishing an important difference with the previous versions that contemplated five factors, with a good internal consistency and having as a central axis the stress produced by the misfit between the person and his/her environment. Thus, the instrument allowed for the creations of a scale for measuring technostress in Chilean university students (TS4US) based on the following factors: Abilities-Demands Techno-Educational (ADTE), Needs-Supplies Resources (NSR), and Person-People Factor (PPF). It will also allow for a continuation of the measurements of technostress in a local context, increasing the studies on this topic already carried out in different actors of the Chilean educational system.

Although all the parameters that ensure the quality of the sample size were met in this study and the sample exceeded the small sample cut-off for a factor analysis [43,44,45,46,47,48,49,50,51,52,53,54,55,56,57,58,59,60,61,62,63,64,65,66], a limitation of this work was not having a larger sample, which we will address after the validation of this psychometric instrument. On the other hand, the article has been limited only to the validity analysis on the psychometric measurement scale studied, without analyzing other dimensions. Also, future research lines will allow for a series of local studies on technostress in the Chilean educational system [18,19,25,67] to be completed, as well as for extensive measurements to be developed on technostress in university students, and further work relating technostress with other aspects, such as techno-addiction, cyberbullying, transformation of learning processes and engagement to educational work, and stimulating ‘learning by doing’ [5,6,68,69,70].

## Figures and Tables

**Table 1:** Validation and reliability reported in previous articles and parameters.

Article	Country	Sample	Method	Factors	MIF	χ^2^/df	RMSEA	AGFI	GFI	CFI	RFI	NFI	NNFI	RMSR
Wang et al. [21]	China	343	EFA/CFA	5	4	2.06 *	0.06 *	NR	NR	0.95 *	NR	0.91 *	NR	NR
Penado-Abilleira et al. [38]	Spain	1744	EFA/CFA	5	3	NR	NR	0.994 **	0.995 **	NR	0.993 **	0.994 **	NR	0.054 *
Schermelleh-Engel et al. [46]	Parameters	≥200	Good fit	-	NR	≥0 ≤2	≤0.05	≥0.90 ≤1.00	≥0.95 ≤1.00	≥0.97 ≤1.00	>0.90 ^+^	≥0.95 ≤1.00	≥0.97 ≤1.00	<0.05 ^++^
			Acceptable fit	-	≥3	>2 ≤3	>0.05 ≤0.08	≥0.85 <0.90	≥0.90 <0.95	≥0.95 <0.97	NR	≥0.90 <0.95	≥0.95 <0.97	≥0.05 ≤0.08 ^++^

NR: not reported. ** Good fit; * acceptable fit. ^+^ indicated in Penado-Abilleira et al. [38]. ^++^ indicated in Kalkan et al. [47].

**Table 2:** Participant sample characterization.

Variable	Category/Level	Frequency	Percentage
University Type	State University	71	33.5%
	Private University	141	66.5%
Educational Level	Postgraduate	10	4.7%
	Undergraduate	202	95.3%
Educational Journey	Daytime (synchronous)	129	60.8%
	Evening (synchronous)	30	14.2%
	Weekends (synchronous)	6	2.8%
	Online (asynchronous)	47	22.2%
Age Level	Less than 20 years old	67	31.6%
	21 to 30 years	116	54.7%
	31 to 40 years old	22	10.4%
	41 to 50 years old	5	2.4%
	51 to 60 years old	1	0.5%
	60 years and older	1	0.5%
	Average age	N/A	N/A
Job Condition (Chilean census standard)	I am looking for a job for the first time (unemployed)	13	6.1%
	I am unemployed (unemployed)	21	9.9%
	I am physically or mentally unable to work (unemployed)	3	1.4%
	I am exclusively studying (not working)	114	53.8%
	I am busy	56	26.4%
	I am not interested in working	5	2.4%
Gender	Female	151	71.2%
	Male	61	28.8%

**Table 3:** Communalities.

Variable	1	2	3	4	6	7	8	9	11	12	13	14	15	16	17	18	19	20	21
Initial	0.639	0.670	0.707	0.713	0.669	0.610	0.721	0.581	0.505	0.744	0.633	0.497	0.465	0.480	0.638	0.494	0.462	0.468	0.470
Extraction	0.568	0.573	0.718	0.833	0.661	0.641	0.742	0.604	0.427	0.755	0.655	0.449	0.405	0.497	0.683	0.477	0.524	0.563	0.525

**Table 4:** Exploratory Factor Analysis for 4 factors.

KMO and Bartlett’s Test					
Kaiser-Meyer-Olkin Measure of Sampling Adequacy.					0.897
Bartlett’s Test of Sphericity			Approx. Chi-Square		2432.170
			Degree of freedom		171
			Significance		0.000
Pattern Matrix ^a^					
ID	Variable	Factor			
		1	2	3	4
ADO1	VAR_01	0.678			
ADO2	VAR_02	0.534			
ADO3	VAR_03				0.692
ADO4	VAR_04				0.864
NSO1	VAR_06		0.783		
NSO2	VAR_07		0.739		
NSO3	VAR_08		0.902		
NSO4	VAR_09		0.774		
ADT3	VAR_12	0.603			
ADT4	VAR_13	0.702			
NST1	VAR_14		0.623		
NST2	VAR_15		0.544		
NST3	VAR_16	0.729			
NST4	VAR_17	0.709			
NST5	VAR_18	0.651			
PPF1	VAR_19			0.695	
PPF2	VAR_20			0.712	
PPF3	VAR_21			0.628	
% of Variance		41.176	10.083	4.655	3.561
Cumulative %		41.176	51.259	55.915	59.476
Factor Correlation Matrix ^b^					
Factor		1	2	3	4
1		1.000			
2		0.458	1.000		
3		0.369	0.492	1.000	
4		0.553	0.367	0.314	1.000

^a^ Extraction Method: Unweighted Least Squares. Rotation Method: Oblimin with Kaiser Normalization. Rotation converged in 8 iterations. ^b^ Extraction Method: Unweighted Least Squares. Rotation Method: Oblimin with Kaiser Normalization.

**Table 5:** Communalities.

Variable	1	2	3	4	6	7	8	9	11	12	13	14	15	16	17	18	19	20	21
Initial	0.639	0.670	0.707	0.713	0.669	0.610	0.721	0.581	0.505	0.744	0.633	0.497	0.465	0.480	0.638	0.494	0.462	0.468	0.470
Extraction	0.541	0.581	0.550	0.503	0.650	0.638	0.746	0.602	0.424	0.768	0.647	0.432	0.402	0.414	0.647	0.429	0.509	0.540	0.519

**Table 6:** Exploratory Factor Analysis for 3 factors.

Pattern Matrix ^a^				
ID	Variable	Factor		
		1	2	3
ADO1	VAR_01	0.724		
ADO2	VAR_02	0.733		
ADO3	VAR_03	0.727		
ADO4	VAR_04	0.691		
NSO1	VAR_06		0.789	
NSO2	VAR_07		0.746	
NSO3	VAR_08		0.916	
NSO4	VAR_09		0.776	
ADT2	VAR_11	0.652		
ADT3	VAR_12	0.887		
ADT4	VAR_13	0.831		
NST1	VAR_14		0.614	
NST2	VAR_15		0.539	
NST3	VAR_16	0.649		
NST4	VAR_17	0.745		
NST5	VAR_18	0.620		
PPF1	VAR_19			0.683
PPF2	VAR_20			0.720
PPF3	VAR_21			0.624
% of Variance		40.952	9.937	4.583
Cumulative %		40.952	50.890	55.473
Factor Correlation Matrix ^b^				
Factor		1	2	3
1		1.000		
2		0.536	1.000	
3		0.447	0.503	1.000

^a^ Extraction Method: Unweighted Least Squares. Rotation Method: Oblimin with Kaiser Normalization. Rotation converged in 5 iterations. ^b^ Extraction Method: Unweighted Least Squares. Rotation Method: Oblimin with Kaiser Normalization.

**Table 7:** Confirmatory Factor Analysis for 3 factors.

Rotated Loading Matrix					
Previous	TS4US	Variable	Factor		
			1	2	3
ADO1	ADTE01	VAR_01			0.732
ADO2	ADTE02	VAR_02			0.745
ADO3	ADTE03	VAR_03			0.734
ADO4	ADTE04	VAR_04			0.696
NSO1	NSR01	VAR_06	0.794		
NSO2	NSR02	VAR_07	0.747		
NSO3	NSR03	VAR_08	0.924		
NSO4	NSR04	VAR_09	0.779		
ADT2	ADTE05	VAR_11			0.657
ADT3	ADTE06	VAR_12			0.894
ADT4	ADTE07	VAR_13			0.841
NST1	NSR05	VAR_14	0.612		
NST2	NSR06	VAR_15	0.535		
NST3	ADTE08	VAR_16			0.657
NST4	ADTE09	VAR_17			0.749
NST5	ADTE10	VAR_18			0.629
PPF1	PPF1	VAR_19		0.714	
PPF2	PPF2	VAR_20		0.760	
PPF3	PPF3	VAR_21		0.652	
% of Variance			43.204	12.112	6.991
Cumulative %			43.204	55.316	62.307
Factor Correlation Matrix					
Factor			1	2	3
1			1.000		
2			0.510	1.000	
3			0.558	0.545	1.000

**Table 8:** Validation and reliability reported in previous articles and parameters.

Article	Country	Sample	Method	Factors	MIF	χ^2^/df	RMSEA	AGFI	GFI	CFI	RFI	NFI	NNFI	RMSR
Wang et al. [21]	China	343	EFA/CFA	5	4	2.06 *	0.06 *	NR	NR	0.95 *	NR	0.91 *	NR	NR
Penado-Abilleira et al. [38]	Spain	1744	EFA/CFA	5	3	NR	NR	0.994 **	0.995 **	NR	0.993 **	0.994 **	NR	0.054 *
Proposed Model	Chile	212	EFA/CFA	3	4	NR	0.072 *	0.986 **	0.990 **	0.979 **	NR	NR	0.970 **	0.047 **
Schermelleh-Engel et al. [46]	Parameters	≥200	Good fit	-	NR	≥0 ≤2	≤0.05	≥0.90 ≤1.00	≥0.95 ≤1.00	≥0.97 ≤1.00	>0.90 ^+^	≥0.95 ≤1.00	≥0.97 ≤1.00	<0.05 ^++^
			Acceptable fit	-	≥3	>2 ≤3	>0.05 ≤0.08	≥0.85 <0.90	≥0.90 <0.95	≥0.95 <0.97	NR	≥0.90 <0.95	≥0.95 <0.97	≥0.05 ≤0.08 ^++^

NR: not reported. ** Good fit; * acceptable fit. ^+^ indicated in Penado-Abilleira et al. [38]. ^++^ indicated in Kalkan et al. [47].

**Table 9:** Reliability statistics.

Factor	Factor Name	Cronbach’s Alpha	Cronbach’s Alpha Base on Standardized Items	Number of Items
1	Needs-Supplies Resources, NSR	0.887	0.886	6
2	Person-People Factor, PPF	0.753	0.754	3
3	Abilities-Demands Techno-Educational, ADTE	0.921	0.922	10
Total	TS4US scale	0.925	0.925	19

## Data Availability

Data Availability in Supplementary Materials.

## Supplementary Materials

The following supporting information can be downloaded at: https://www.mdpi.com/article/10.3390/ijerph192114493/s1, Table S1: TS4US_data_22_var.

## Appendix A. Applied Questionnaire (In Spanish of Current Use in Chile)

**Table A1:** Applied Technostress Questionnaire.

Previous	Variable	Questions (In Chilean Spanish)
ADO1	VAR_01	Me resulta difícil satisfacer las demandas de mi universidad con respecto al uso de las tecnologías de información
ADO2	VAR_02	Me resulta difícil implementar con eficacia las indicaciones de mi universidad sobre el uso de las tecnologías de información
ADO3	VAR_03	Mi capacidad actual es insuficiente para implementar las indicaciones de mi universidad sobre el uso de las tecnologías de información
ADO4	VAR_04	Mis habilidades actuales son insuficientes para implementar las indicaciones de mi universidad sobre el uso las tecnologías de la información
ADO5	VAR_05	Me resulta difícil organizar mi horario de estudio actual para cumplir con las indicaciones de mi universidad sobre sobre el uso de las tecnologías de información
NSO1	VAR_06	Mi universidad no me brinda suficiente inducción para usar las tecnologías de información de manera efectiva en mis actividades académicas
NSO2	VAR_07	Mi universidad no me brinda incentivos suficientes para utilizar las tecnologías de información de manera efectiva en mis actividades como estudiante
NSO3	VAR_08	La inducción desarrollada por mi universidad no es muy útil para lograr un uso efectivo de las tecnologías de información
NSO4	VAR_09	En mi universidad no existe una cultura que fomente el uso de herramientas innovadoras como las tecnologías de información
ADT1	VAR_10	Me siento presionado/a para usar las tecnologías de información de manera efectiva en mis trabajos universitarios
ADT2	VAR_11	Me resulta difícil utilizar las tecnologías de información de manera efectiva debido al poco tiempo y esfuerzo que le dedico
ADT3	VAR_12	Me resulta difícil hacer frente a las altas demandas de las tecnologías de información con mi capacidad actual
ADT4	VAR_13	Me resulta difícil ponerme al día con los rápidos cambios de las tecnologías de información
NST1	VAR_14	Las tecnologías de información en mi universidad no son efectivas para ayudarme a aumentar mi desempeño académico
NST2	VAR_15	Las tecnologías de información en mi universidad no son muy importantes
NST3	VAR_16	Estoy abrumado/a por la gran variedad de tecnologías de información que se utilizan en mi universidad
NST4	VAR_17	Las diversas tecnologías de información complican mi proceso de toma de decisiones académicas
NST5	VAR_18	Me molesta el uso excesivo de las tecnologías de información en mi universidad
PPF1	VAR_19	No tengo el apoyo suficiente de mis compañeros para el uso de las tecnologías de información
PPF2	VAR_20	Mis compañeros no son positivos con respecto al uso innovador de las tecnologías de información en mi universidad
PPF3	VAR_21	No tengo un equipo con el que colaborar para encontrar una forma eficaz de usar las tecnologías de información en mis actividades como estudiante universitario
PPF4	VAR_22	A menudo siento que estoy solo explorando el uso innovador de las tecnologías de la información

## Appendix B. Definitive TS4US Scale (Presented in Chilean Spanish)

**Table A2:** TS4US Questionnaire.

TS4US	Variable	Questions (In Chilean Spanish)
ADTE01	VAR_01	Me resulta difícil satisfacer las demandas de mi universidad con respecto al uso de las tecnologías de información
ADTE02	VAR_02	Me resulta difícil implementar con eficacia las indicaciones de mi universidad sobre el uso de las tecnologías de información
ADTE03	VAR_03	Mi capacidad actual es insuficiente para implementar las indicaciones de mi universidad sobre el uso de las tecnologías de información
ADTE04	VAR_04	Mis habilidades actuales son insuficientes para implementar las indicaciones de mi universidad sobre el uso las tecnologías de la información
ADTE05	VAR_05	Me resulta difícil organizar mi horario de estudio actual para cumplir con las indicaciones de mi universidad sobre sobre el uso de las tecnologías de información
ADTE06	VAR_11	Me resulta difícil utilizar las tecnologías de información de manera efectiva debido al poco tiempo y esfuerzo que le dedico
ADTE07	VAR_12	Me resulta difícil hacer frente a las altas demandas de las tecnologías de información con mi capacidad actual
ADTE08	VAR_13	Me resulta difícil ponerme al día con los rápidos cambios de las tecnologías de información
ADTE09	VAR_16	Estoy abrumado/a por la gran variedad de tecnologías de información que se utilizan en mi universidad
ADTE10	VAR_17	Las diversas tecnologías de información complican mi proceso de toma de decisiones académicas
ADTE11	VAR_18	Me molesta el uso excesivo de las tecnologías de información en mi universidad
NSR01	VAR_06	Mi universidad no me brinda suficiente inducción para usar las tecnologías de información de manera efectiva en mis actividades académicas
NSR02	VAR_07	Mi universidad no me brinda incentivos suficientes para utilizar las tecnologías de información de manera efectiva en mis actividades como estudiante
NSR03	VAR_08	La inducción desarrollada por mi universidad no es muy útil para lograr un uso efectivo de las tecnologías de información
NSR04	VAR_09	En mi universidad no existe una cultura que fomente el uso de herramientas innovadoras como las tecnologías de información
NSR05	VAR_14	Las tecnologías de información en mi universidad no son efectivas para ayudarme a aumentar mi desempeño académico
NSR06	VAR_15	Las tecnologías de información en mi universidad no son muy importantes
PPF1	VAR_19	No tengo el apoyo suficiente de mis compañeros para el uso de las tecnologías de información
PPF2	VAR_20	Mis compañeros no son positivos con respecto al uso innovador de las tecnologías de información en mi universidad
PPF3	VAR_21	No tengo un equipo con el que colaborar para encontrar una forma eficaz de usar las tecnologías de información en mis actividades como estudiante universitario
PPF4	VAR_22	A menudo siento que estoy solo explorando el uso innovador de las tecnologías de la información
